# Genetic Optimization of Energy- and Failure-Aware Continuous Production Scheduling in Pasta Manufacturing

**DOI:** 10.3390/s19020297

**Published:** 2019-01-13

**Authors:** Ke Shen, Toon De Pessemier, Xu Gong, Luc Martens, Wout Joseph

**Affiliations:** 1 Department of Information Technology, Ghent University/IMEC, Technologiepark 126, 9052 Ghent, Belgium; Toon.DePessemier@UGent.be (T.D.P.); Luc1.Martens@ugent.be (L.M.); Wout.Joseph@UGent.be (W.J.); 2 Huawei Technologies, Songshan Lake Technology Park, Dongguan 523808, China; xu.gong@outlook.com

**Keywords:** genetic algorithm, continuous production scheduling, energy and failure management, pasta manufacturing

## Abstract

Energy and failure are separately managed in scheduling problems despite the commonalities between these optimization problems. In this paper, an energy- and failure-aware continuous production scheduling problem (EFACPS) at the unit process level is investigated, starting from the construction of a centralized combinatorial optimization model combining energy saving and failure reduction. Traditional deterministic scheduling methods are difficult to rapidly acquire an optimal or near-optimal schedule in the face of frequent machine failures. An improved genetic algorithm (IGA) using a customized microbial genetic evolution strategy is proposed to solve the EFACPS problem. The IGA is integrated with three features: Memory search, problem-based randomization, and result evaluation. Based on real production cases from Soubry N.V., a large pasta manufacturer in Belgium, Monte Carlo simulations (MCS) are carried out to compare the performance of IGA with a conventional genetic algorithm (CGA) and a baseline random choice algorithm (RCA). Simulation results demonstrate a good performance of IGA and the feasibility to apply it to EFACPS problems. Large-scale experiments are further conducted to validate the effectiveness of IGA.

## 1. Introduction

Energy modeling for fabrication processes and energy-aware production scheduling are fundamental issues for energy management in manufacturing systems. The former is in the scope of energy efficiency (EE) [1], investigating opportunities for energy saving in a production process. The latter is in the field of demand response (DR) [2], taking into account volatile energy prices and the power profiles of machines. In literature, energy consumption is regularly tackled as one objective of scheduling, engaged in the optimization model with other objectives, including makespan [3], reliability [4], tardiness [5], or labor cost [6]. Especially in continuous manufacturing systems, such as steel-making [7] and textile dyeing [8], although these processes are extremely energy-sensitive, energy saving has a limited contribution to the optimization compared to other objectives.

Certainly, energy management cannot be ignored for energy-sensitive continuous production scheduling. Among other objectives, reliability is one of the most difficult to achieve. A provided schedule is often faced with uncertainties in actual production, preventing it from being executed as planned, where failure uncertainty is considered the most significant uncertainty in production scheduling [9]. However, uncertainties are usually not taken into consideration during the execution of schedules [10], since the reliability of the environment is hard to measure. Stochastic modeling is frequently used in literature to define and reflect reliability [11], of which the accurate prediction is difficult because of the massively multivariate production environment. Additionally, stochastic methods are highly dependent on historical records, but the root cause (needed to effectively prevent uncertainties) are not easily found by only looking at statistics [12]. In literature, failure uncertainty is considered a constraint in most cases because of the aforementioned difficulties in quantitatively handling it as an objective. Although failure reduction appears to be more difficult than the broadly studied energy saving, this paper presents an example where modeling techniques for the latter can be tailored to assist with the former.

The investigated problem in the research is an energy- and failure-aware continuous production scheduling problem (EFACPS). Although the continuous scheduling problem at the factory level is often considered a flexible flow shop (FFS) scheduling problem [13], this paper focuses on EFACPS on a single machine at the unit process level. After reviewing current research of energy-aware scheduling and failure-related scheduling (see Section 2), the investigated problem was formulated as a centralized combinatorial optimization model. Since energy saving and failure reduction are always separately studied in literature, this model is an early effort to combine both energy and failure modeling in production scheduling.

The studied EFACPS problem is deduced as NP-hard. This paper also provides an improved genetic algorithm (IGA) using a customized microbial genetic evolution strategy [14] to solve it. The IGA is introduced with three integrated features: Memory search, problem-based randomization, and result evaluation. These features enhance its convergence speed without trapping into local optima. Compared with a conventional genetic algorithm (CGA) and a baseline random choice algorithm (RCA), whose pseudo codes are available in this study, the proposed IGA can obtain a near-optimal schedule for real EFACPS problems in a shorter time. Monte Carlo simulations (MCS) using actual industrial data evaluated the performances of the algorithms, indicating that IGA has the best performance.

The main contributions of this paper are as follows: (1) The energy- and failure-aware production scheduling model introduced in this paper puts forward an exceptional perspective combining energy and failure modeling; (2) failure reduction is demonstrated as a possible objective of production scheduling, which was normally considered as a criterion in literature; (3) an IGA using a customized microbial evolution strategy is proposed to solve the EFACPS problem, integrated with new features; (4) the performances of IGA, CGA and RCA were investigated using MCS based on actual production data from a large pasta manufacturer; (5) discussions on parameter resolution for the model and for the algorithms are provided.

The remainder of this paper is organized as follows. Section 2 provides a literature review revealing the problem. In Section 3, the primary notation, mathematical formulation, and the optimization model of the problem are introduced. In Section 4, IGA, CGA, and RCA are presented to solve the NP-hard problem. Section 5 investigates different cases derived from empirical records of the company. The performance of the provided IGA is evaluated from the perspectives of convergence speed and complexity. Discussions on parameter preferences in solving the real EFACPS are also held in this section. Section 6 finally draws the related conclusions.

## 2. Literature Review

The studied issues in this paper involve specific production scheduling problems with energy- or failure-related objectives. Methodologies for detecting and handling failures in corresponding fields are also inspected.

### 2.1. Energy-Aware Scheduling

Energy-aware scheduling was broadly studied in recent research. Multiobjective optimization models were proposed in literature, which were often deduced or proved as NP-hard. Heuristics were frequently used to search for near-optimal solutions.

Gong et al. [15] formulated a mixed-integer linear programming (MILP) mathematical model for energy-aware production scheduling on a single machine, where a finite state machine (FSM) was used for energy modeling of different machine states. A conventional genetic algorithm was implemented to give an energy-efficient solution even at the presence of stochasticity, verified by empirical data from a grinding machine. For 35 jobs in 7 days, the GA took 2593 s to search for a near-optimal schedule. The performance of the algorithm was intended to improve. Jaclason et al. [16] presented a multiobjective nonlinear programming model solved with the nondominated sorted genetic algorithm (NSGA-II), aiming to schedule the use of home appliances based on the price of electricity in real-time (RTP). Statistical analysis indicated that NSGA-II has a better performance than a random GA. Details of the adaptation of the algorithm to the actual problem were not mentioned. István et al. [17] investigated the design of robust production scheduling proactively guaranteeing the energy consumption limit with uncertainty scenarios. Two exact (branch-and-bound and logic-based benders decomposition) and one heuristic algorithm (tabu search) were used to find an optimal permutation of given operations. Afterwards, a pseudo-polynomial algorithm was proposed to find the optimal robust schedule for that permutation. The master problem as a MILP model was solved by an existing optimizer, Gurobi. Guo et al. [5] addressed a flow shop scheduling problem minimizing energy consumption and tardiness penalties with the constraints of machine-state-dependent setup time. Fuzzy numbers were used to determine the impact of uncertainty. A GA with better performance than random GA was introduced, but the genetic operations were problem-specified and hard to adapt to other scenarios. Guillaume et al. [3] studied energy-aware scheduling of task execution on multiprocessors. The NP-hardness of the problem was proven; afterwards, possible solutions were discussed. Multiple heuristics were examined to get a near-optimal schedule, where the best heuristic was defined as the one with the minimum value of the objective function. If the scenario changes, the proposed heuristic might not remain efficient.

Research on energy-aware scheduling has provided multiple modeling techniques and algorithms for relatively efficient solutions. Further reviews of failure-related scheduling attempts to discover commonalities in problem modeling and algorithm design are needed.

### 2.2. Failure-Related Scheduling

Stochastic modeling is used in many studies by assuming or estimating a probability distribution of uncertainty during production periods [18]. These methods highly depend on historical records to fit the distribution. Robust scheduling does not have these kinds of limits, since no probability distribution of uncertainty is needed [19]. Instead of modeling uncertainties using stochastic approaches, it is designed to “absorb” uncertainty [20].

Li et al. [21] investigated a workload scheduling problem in cloud data centers. Reliability indexes were defined and labeled using worst-fit and best-fit strategies; afterwards, algorithms were designed to make the best use of the most reliable and powerful servers in data centers. The proposed method benefited from logs of data centers, while in other scenarios, detailed records of failure are limited. Jiang et al. [22] studied production scheduling with uncertain processing times for the steel-making continuous casing problem. An estimation distribution algorithm (EDA) using variate processing time as the uncertainty factor was proposed, also firmly driven by records of processing times. Wang et al. [23] worked on production scheduling of precast components in the face of uncertain workflow. The discrete event simulation (DES) was used to evaluate the feasible options using genetic algorithms. Uncertain processing time and complex resource constraints were taken into account in simulations. A trade-off was achieved between on-time delivery and minimum production cost. The proposed approach was implemented in the simulation software ARENA on a commercial optimization engine. Lu et al. [24] assumed a machine breakdown following the Weibull failure function in a maintenance planning production scheduling problem. A bi-objective genetic algorithm was used for saving energy and meeting deadlines, with preventive maintenance integrated into production orders. However, Weibull distribution is not always an ideal model for failures in other scenarios. Guo et al. [25] dealt with a multiobjective production scheduling problem at the factory level, where uncertainties were described as continuous or discrete random variables. Factors including completion time and start time of production processes were derived using probability theory. A linear model based on the concept of satisfactory level was used to represent the impact of uncertainty. Stochastic optimization with such a basic model is not sufficient for complex systems. Drwal et al. [26] provided a polynomial time optimization algorithm for the problem of scheduling jobs with uncertain completion due-dates on a single machine. Jobs were supposed to have equal weights and uncertain due-date intervals were randomly generated. For more general problems with different weights of jobs, the research conjectured the problem to be NP-hard and presented heuristics algorithms as possible approaches. Shown in experimental results, computational complexity increased significantly in some cases. Ghezail et al. [27] introduced graphical representations of scheduling solutions to model the robustness of the schedule and the impacts of unexpected disruptions. This method avoided restrictive quantitative approaches used for robust scheduling, based on an assumption that the initial order of jobs was always respected.

In literature, there were many differences between energy and failure modeling. Simulation methods were frequently used to evaluate customized representations of failure uncertainty. Similar to energy-aware scheduling, failure-related scheduling problems were regularly deduced as NP-hard, where heuristics were habitually applied.

To sum up, the scheduling problem has always been formulated in the abstract as ‘find from a Set *S* of candidate schedules a subset *T* that satisfies some criterion *C* and minimizes an objective function *f*’. Heuristic methods are commonly applied to solve this combinatorial optimization problem [28].

## 3. Problem Formulation

The EFACPS problem studied in this paper was formulated by investigating three subproblems, including ordinary production scheduling, energy-aware scheduling, and failure-aware scheduling. Afterwards, a centralized combinatorial optimization model is proposed.

### 3.1. Notation

The description of parameters used in this section is shown in Table 1.

#### 3.1.1. Production Scheduling

Continuous production scheduling on a single machine is defined with the following constraints:

- The machine is kept busy if there are remaining jobs to finish.
- The machine has a product-type-dependent production speed.
- The process of a job starts when it is charged on the machine and ends when it is unloaded.
- A new job is charged to the machine immediately after the end of the previous job.
- Each job has a specific product type and a target quantity.
- Preemption is not allowed, since changeover of jobs causes excessive waste.

The scheduling model is constructed from the fundamental objects of the problem. Waiting jobs are denoted using set $J=\{j_{1},\;j_{2},\;\ldots ,\;j_{i},\;\ldots ,\;j_{N}\}$, where *N* is the total number. A job $j_{i}$ has two attributes: Product type $p_{i}$ and objective quantity $q_{i}$. $P=\{p_{1},\;p_{2},\;\ldots ,\;p_{M}\}$ is the set of possible product types. The production speed of a product with type $p_{i}$ is defined as $v_{p_{i}}$, where $p_{i}\in P$. Before planning a schedule, all aforementioned parameters (J,P, and $p_{i}$, $v_{p_{i}}$, $q_{i}$ of each job $j_{i}$) are available and remain constant during production. Such a problem is classified as an offline scheduling problem [29].

The production duration of job $j_{i}$ is denoted as $t_{i}$ and calculated using Equation (1). The start timestamp and end timestamp of $j_{i}$ are defined using $T_{st_{i}}$ and $T_{ed_{i}}$, respectively.

(1) $$t_{i}=q_{i}/v_{p_{i}}$$

The following time constraints make sure that preemption is prohibited and jobs are executed respecting the scheduled sequence:

(2) $$T_{st_{i}}<T_{ed_{i}},\;i\in \left[1,2,\ldots ,N\right]$$

(3) $$T_{ed_{i}}\leq T_{st_{i+1}},\;i\in \left[1,2,\ldots ,N\right]$$

#### 3.1.2. Energy-Aware Scheduling

Either energy or failure management in actual production is extremely complicated due to machine and product characteristics [9]. The energy charging policy used in this study is real-time pricing (RTP) with hourly changed electricity price [6].

A job $j_{i}$ is processed on the machine with four stages: Preparing, Preprocessing, Working and Discharging, denoted as $S=\{s_{1},\;s_{2},\;s_{3},\;s_{4}\}$. The durations of each stage of job $j_{i}$ are represented using the set $T_{s_{i}}=\left\{t_{s_{1i}},\;t_{s_{2i}},\;t_{s_{3i}},\;t_{s_{4i}}\right\}$ with a constraint in Equation (4). The machine energy consumption $E_{i}$ during the production of $j_{i}$ is calculated using Equation (5), where $P_{s}$ is the power consumption of stage *s*.

(4) $$\forall t\in T_{s_{i}},\;t>0,\;i\in \left[1,2,\ldots ,N\right]$$

(5) $$E_{i}=\sum_{s\in S_{i}}\sum_{t\in T_{s_{i}}}P_{s}\cdot t$$

**Figure 1:**
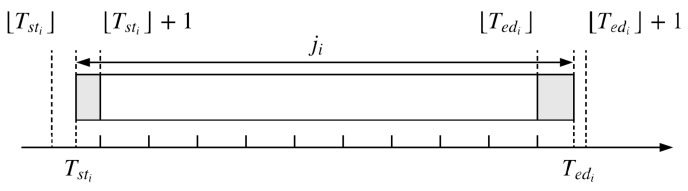
Calculation of energy cost for processing job $j_{i}$.

With RTP, the energy cost $C_{E_{i}}$ to process the job $j_{i}$ can be derived. First, we designed a column vector $D_{i}$ of size $N_{D_{i}}$ to restore useful price information during the production period. Afterwards, $E_{i}$ was discretized into a line vector of size $N_{D_{i}}$. Each element $e_{ij}$ represents energy consumption in the time period between $T_{i}$ and $T_{j}$. This process is illustrated in Figure 1 and Equations (6)–(9).

(6) $$N_{D_{i}}=\left\lfloor T_{ed_{i}}\right\rfloor -\left\lfloor T_{st_{i}}\right\rfloor +1$$

(7) $$D_{i}={\left[\begin{array}{c} d_{\left\lfloor T_{st_{i}}\right\rfloor },\ldots ,d_{\left\lfloor T_{ed_{i}}\right\rfloor } \end{array}\right]}^{T}$$

(8) $$E_{i}=\left[\begin{array}{c} e_{T_{st_{i}}\lfloor T_{st_{i}}\rfloor +1},e_{\lfloor T_{st_{i}}\rfloor +1\lfloor T_{st_{i}}\rfloor +2},\ldots ,e_{\left\lfloor T_{ed_{i}}\right\rfloor T_{ed_{i}}} \end{array}\right]$$

(9) $$C_{E_{i}}=E_{i}\cdot D_{i}$$

In Figure 1, the *head* of $j_{i}$ has the energy cost of $d_{\left\lfloor T_{st_{i}}\right\rfloor }$, starting from $T_{st_{i}}$, ending at $\lfloor T_{st_{i}}\rfloor +1$. The *tail* of $j_{i}$ has the energy cost of $d_{\left\lfloor T_{ed_{i}}\right\rfloor }$, starting from $\left\lfloor T_{ed_{i}}\right\rfloor$, ending at $T_{ed_{i}}$. The *body* of $j_{i}$ has costs $\left[d_{\lfloor T_{st_{i}}\rfloor +1},\;\ldots ,\;d_{\lfloor T_{ed_{i}}\rfloor -1}\right]$, starting from $\lfloor T_{st_{i}}\rfloor +1$, ending at $\left\lfloor T_{ed_{i}}\right\rfloor$.

#### 3.1.3. Failure-Aware Scheduling

The taxonomy of failure awareness in this paper was inspired by Reference [30], which classifies maintenances by their influences on health states of the machine. The set $H=\{h_{1},\;h_{2},\;\ldots ,\;h_{F}\}$ describes machine health states in ascending order of failure rate: $h_{0}$ is the initial health state and $h_{F}$ is the failure state. There are two kinds of possible maintenance actions in the system. $M_{Rr}$ represents real-time response maintenances performed by workshop operators. These actions are carried out during the processing of a job $j_{i}$, making the machine quickly recover from the blockage but slowly fall into a worse health state. $M_{Re}$ represents replacement maintenance actions, such as replacing vulnerable parts or repairing machine components, after which the machine health state will be restored to a better health state. $M_{Re}$ are immediately performed after machine failure or are planned on fixed days.

Before modeling the failure uncertainty, background information of the EFACPS problem needs to be clarified: (1) Raw materials of a job will be lost if machine failure happens after the job charged on the machine; (2) maintenances are organized on fixed days on the production line; (3) compared to the cost of wasted raw materials, the cost of maintenances is negligible.

A schedule π is denoted as $\pi =\{W_{1},\;M_{1},\;W_{2},\;M_{2},\;\ldots ,\;M_{L},\;W_{L+1}\}$, where $M_{i}$ is a maintenance action of type $M_{Re}$ on a fixed date, $W_{i}$ is a batch of jobs between two maintenances, and *L* is the number of maintenances. If a maintenance of type $M_{Re}$ is planned to the time when the machine is processing job $j_{i}$, it is considered to finish before $T_{ed_{i}}$, without postponing the next job. The duration of $M_{Re}$ is neglected under the aforementioned assumptions. $M_{Rr}$ is indirectly used for the problem to distinguish maintenance types.

From the sequence of maintenance actions, the probability of machine failure at a given time is derived. Failure rate r(t) is considered a random variable changing over time *t*, influenced by the effect of maintenance and wear in the machine, satisfying the *memoryless* property that it is based solely on adjacent maintenances [31]. r(t) is presented in Equation (10), where $T_{pm_{i}}$ and $T_{pm_{j}}$ are the time for two adjacent maintenances $M_{i}$ and $M_{j}$. $f_{1}\left(t\right)$ is the average failure rate of time t after maintenance, whereas $f_{2}\left(t\right)$ is the average failure rate of time t before maintenance.

(10) $$r\left(t\right)=r\left(t,T_{pm_{i}},T_{pm_{j}}\right)=\max\left[f_{1}\left(t-T_{pm_{i}}\right),f_{2}\left(T_{pm_{j}}-t\right)\right]$$

Suppose there are *k* hours between two adjacent maintenances. A vector *I* of size *k* is provided to estimate the influence of a maintenance, shown in Equation (11), where $b_{j}$ represents machine failure rate of the *j*th hour after the first maintenance.

(11) $$B=\left[\begin{array}{c} b_{1},\ldots ,b_{k} \end{array}\right]$$

The aforementioned failure model reduces both energy and failure modeling to a similar optimization model. Knowing the hourly dependent failure rate, the failure cost (wasted raw material cost) $C_{F_{i}}$ to process a job $j_{i}$ can be estimated using a close way of calculating $C_{E_{i}}$. A vector $R_{i}$ of size $N_{R_{i}}$ is defined in Equation (12) to represent the failure rate of each hour after raw material is charged on the machine. The calculation of *B* and retrieval of $R_{i}$ from *B* is explained in Section 5.1. If machine failure occurs in the stage Preparing, no failure cost is charged before the stage Preprocessing starts.

(12) $$R_{i}=\left[\begin{array}{c} r\left(\left\lfloor T_{st_{i}}+t_{S_{i1}}\right\rfloor \right),\ldots ,r\left(\left\lfloor T_{ed_{i}}\right\rfloor \right) \end{array}\right]$$

The probability of machine failure occurrence during the production of $j_{i}$ is defined as $P_{Fi}$, calculated using Equation (13), where $r_{i}$ is the *i*th element of $R_{i}$. $C_{F_{i}}$ is calculated using Equation (14), where $u_{p_{i}}$ is the unit raw material cost of $j_{i}$ with product type $p_{i}$.

(13) $$P_{Fi}=1-\prod_{i=1}^{N_{R_{i}}}\left(1-r_{i}\right)$$

(14) $$C_{F_{i}}=P_{Fi}\cdot q_{i}\cdot u_{p_{i}}$$

### 3.2. Optimization Model

The objective of EFACPS is to minimize the overall cost, including failure cost and energy cost. The multiobjective function is presented in Equation (15) where $\varphi_{q}$ is an objective and $\omega_{q}$ is the corresponding weight. The scheduler could set $\omega_{q}$ to 0 in case the related objective $\varphi_{q}$ needs to be ignored.

(15) $$F=\sum_{q}\omega_{q}\varphi_{q}$$

We define the optimization model for the problem in Equation (16), subject to Equations (1)–(14).

(16) $$\min\{\omega_{1}\sum_{i=1}^{N}C_{E_{i}}+\omega_{2}\sum_{i=1}^{N}C_{F_{i}}\}$$

## 4. Method Description

The proposed model has the NP-hardness property according to the following inference: Given a candidate schedule π, no polynomial time verification algorithm is found to accept or reject π as a solution. For the problem of size *N*, its solution domain has the size of N!, since any random permutation of a waiting job sequence leads to a reachable cost. Although the overall cost of π can be calculated in polynomial time using the method introduced in this paper, we could not determine its position in the solution domain. Therefore, the model is not in the class NP and is inherently NP-hard.

A genetic algorithm (GA) is suitable for searching for a solution of an NP-hard problem because of its adaptive global optimization ability [32]. Conventional GA adopts artificial evolution to a population of individuals. Properties are inherited from parents but also altered and mutated. Individuals are selected for a new generation by the desired goal. The provided IGA in this paper extends CGA with the following features, which accelerate the convergence speed and provide an easier ascent towards a global optimum: (1) Memory [33] is introduced to prevent duplicate searches; (2) a customized revolution strategy presented in the microbial genetic algorithm (MGA) [14] is adopted; (3) problem-related random techniques are applied to prevent the algorithm being trapped in local optima; (4) new individuals are evaluated to ensure a significant difference from their parents.

**Figure 2:**
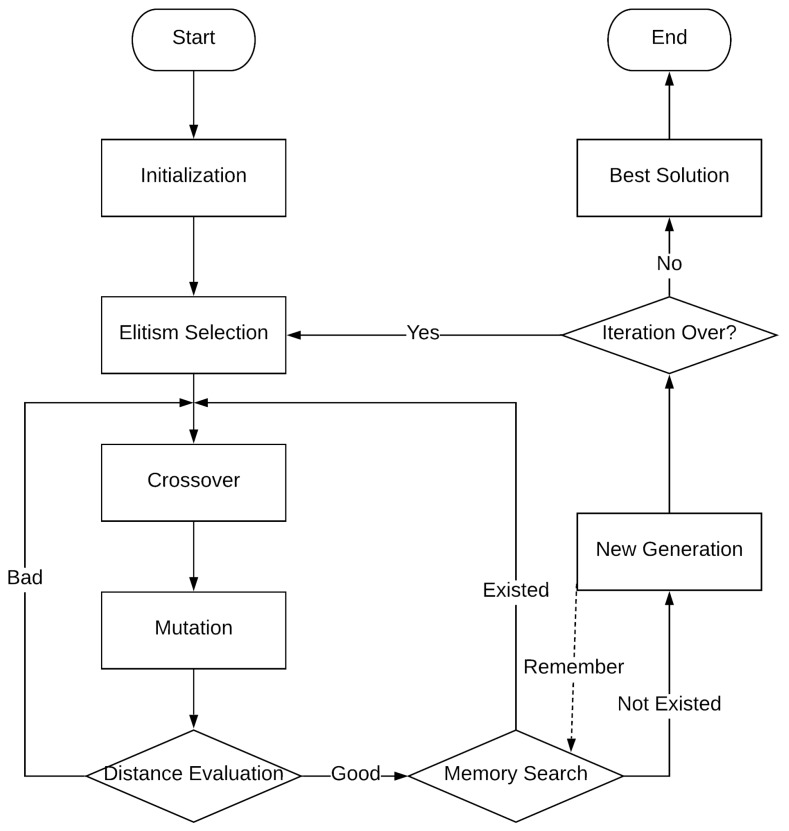
Flowchart of the proposed improved genetic algorithm (IGA).

The procedure of the algorithm is presented in Figure 2 and discussed below.

### 4.1. Initialization

Initial values are starting points in the search space and are highly engaged in influencing the performance of GA. These values also need to meet the constraints of the model. The population size $n_{g}$ is another important problem-based tuning parameter, since there is a threshold between the requirement of computational resources (time and space) in each iteration and the global convergence speed of the algorithm. For EFACPS of *N* waiting jobs, an individual is a candidate schedule encoded with indexes of jobs. A randomly generated vector *I* of size *N* is provided to represent an individual, whose elements are indexes of jobs, implying their execution order.

Randomness tests and resampling techniques could also be performed to ensure the randomness of initial values, which is not the topic of this paper.

### 4.2. Elitism Selection

The CGA stochastically selects more fitted individuals from the current generation. Afterwards, genetic operations (crossover and mutation) are evaluated on these individuals to obtain a new generation. The fitness value is used as a condition for individual selection. The elitism of the current generation is altered by genetic operations.

For EFACPS, the fitness value of an individual is the overall cost of such a candidate schedule, calculated using Equation (16). Since elitism is not reserved in each iteration, the application of CGA (see Algorithm 1) to the problem can lead to no converged evolutions in some iteration. One possible solution is using a buffer to trace the elitism of each iteration as a candidate for global optima. This approach is also used in the RCA (see Algorithm 2). Compared to the provided IGA (see Algorithm 3), such an operation keeps the global convergence but cannot guarantee the local convergence in each iteration.

**Algorithm 1:** Conventional genetic algorithm (CGA).

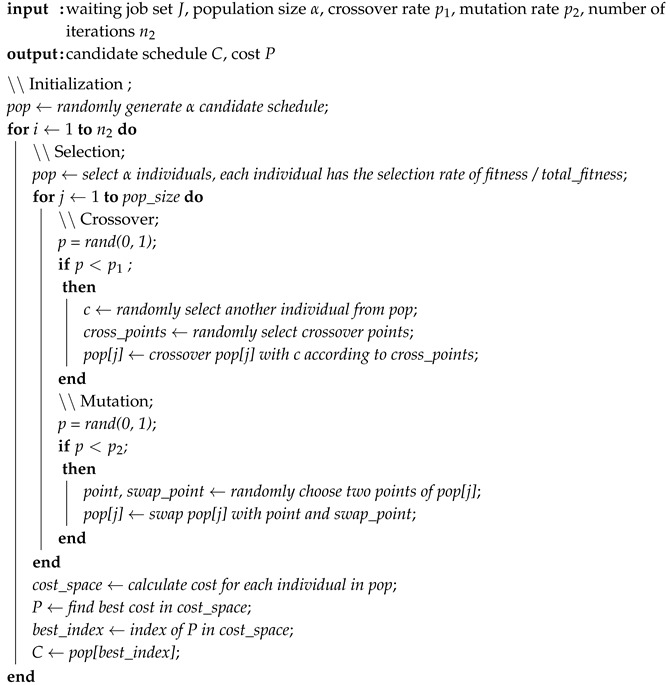



The selection strategy used in the IGA is based on the notation of *winner* and *loser*. Two individuals are randomly chosen from the current generation and sorted by their fitness values, where the *winner* has a smaller total cost. Afterwards, the *winner* is chosen as the elitism and kept identical in the next generation, while the *loser* is modified with the following genetic operations. This process is repeated several times, and each time two children are generated: one is the elitism (same as the *winner*) and the other is the altered *loser*.

**Algorithm 2:** Random choice algorithm (RCA).

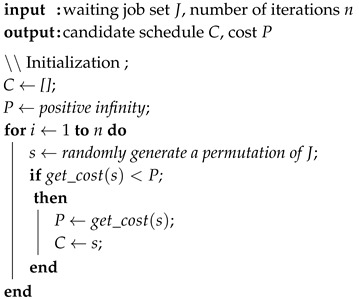



### 4.3. Genetic Operators

**Figure 3:**
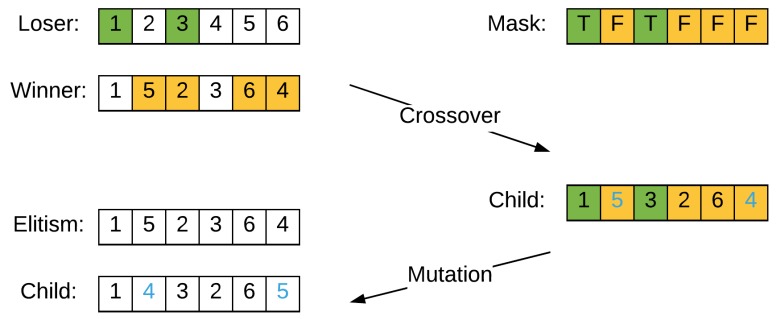
Individual crossover and mutation.

The crossover and mutation processes are explained in Figure 3. With a randomly generated mask, the two selected chromosomes (*winner* and *loser*) generate a new child. Afterwards, a swap is performed on two randomly chosen points of the generated child.

Figure 3 presents crossover and mutation in one iteration on an example of six jobs with indexes {1,2,…,6}. After elitism selection, the *Winner* is [1,5,2,3,4,6] and the *Loser* is [1,2,3,4,5,6]. A *Mask* is generated as [T,F,T,F,F,F]. For the parents {Winner,Loser}, *winner* is kept as the *elitism* in the next iteration. *Child* is generated according to the *mask*: Positions of *loser* marked with false in yellow color inherit values of *winner*. Positions marked with true in green color remain unchanged. Two points of *child* are selected and marked in blue color, on which a swap is performed. The mask is randomly generated and the swap points are randomly chosen.

The hamming distance between the *loser* and the generated child is calculated, which is the number of positions at which the corresponding symbols are different [34]. The aforementioned genetic operations will re-execute if the distance is small, ensuring considerable difference between the *loser* and the child.

**Algorithm 3:** Improved genetic algorithm (IGA).

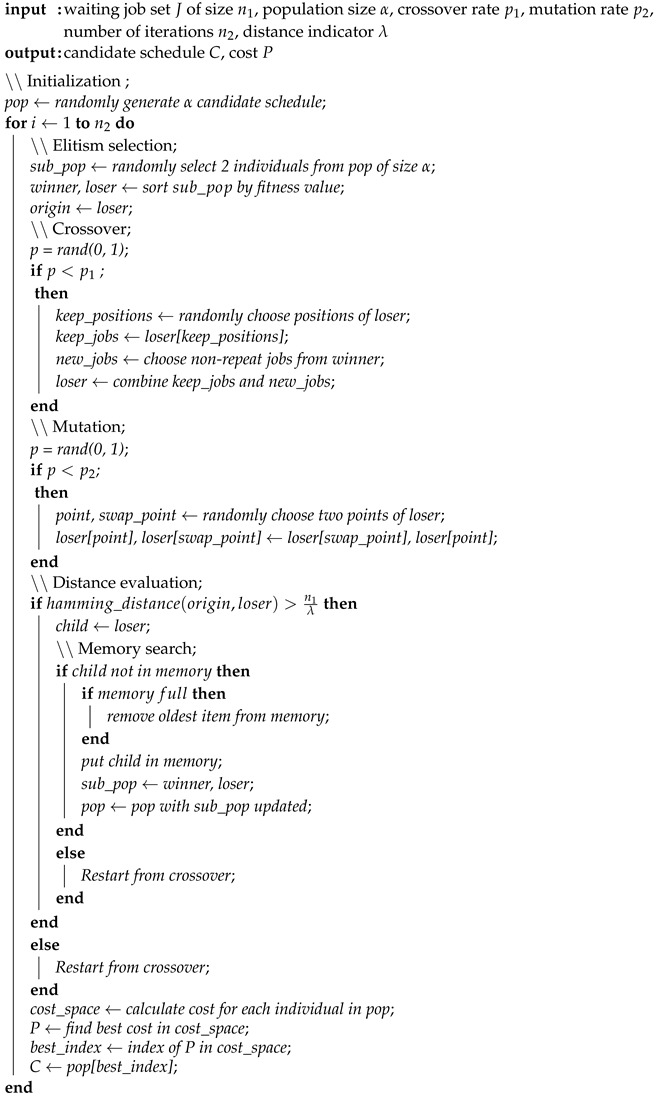



The algorithm also examines the existence of the generated child in the memory space. If the answer is positive, the child will be dropped and generated again to avoid a repeated search. At the end of each iteration, individuals of the current generation, represented using vectors, are saved to a memory space of fixed size. These items are inserted and removed according to the first-in, first-out (FIFO) principle.

## 5. Case Study

Pasta is the 331st most traded and the 1069th most complex merchandise according to the product complexity index (PCI) [35]. In 2014, 14.3 million tons of pasta were produced and traded worldwide, worth 8.4 billion US dollars [36]. Pasta production is both energy-sensitive and quality-focused [37], including four major stages: (1) In the mixing stage, wheat semolina or flour is mixed with water and optional ingredients (egg, salt, vegetable juice, etc.). The mixture is then put through a vacuum dough mixer to produce a homogeneous mass without air bubbles; (2) in the extrusion stage, the extruder pushes the dough through dies to form the shape. Blades of trimmers cut the dough in desired length; (3) in the drying stage, pasta is dried to a desired moisture, giving it a firm shape and a long storage life; (4) in the packaging stage, pasta is packaged into bags or boxes which are ready to transport.

Disruptions in the aforementioned stages cost huge loss of semifinished products. For instance, blockage in the extrusion stage for a relatively long time makes the dough hard to shape or even spoiled. The studied cases in this paper are derived from Soubry N.V., a large pasta manufacturer in Belgium. With the constantly upgrading IoT (Internet of Things) systems, such disturbances in the continuous production process can be better measured and supervised. Empirical data concerning energy consumption and failure measurement are provided by intervention of wireless sensors.

### 5.1. Parameter Resolution

This section presents how to retrieve parameters of the EFACPS model from empirical data saved by the manufacturing execution system (MES) used in the company. MES provides information that helps decision makers to understand the current situation of the shop floor [38]. After preprocessing for intended uses in production scheduling, the following records were examined:

- Order information records (OIR) contain general attributes of orders processed in the job shop, including planning time, objective quantity, product type, and raw material cost.
- Order production records (OPR) contain processing details of orders, including effective time and production speed.
- Machine state records (MSR) contain runtime and downtime periods of the machine in the processing window of each order.
- Maintenance event records (MER) contain dates of maintenance events.

In addition, RTP data were used as the energy price in the experiment, taken from Belpex, the electricity spot market in Belgium [39]. Most of the parameters used in the problem formulation can be found in the aforementioned records, see Table 2. Other parameters which cannot be directly retrieved in those records were divided into two groups: Parameters ($S_{i},\;S_{ik},\;T_{S_{i}},\;t_{S_{ik}},\;P_{s}$) about processing stages and parameters ($B,\;b_{k},\;R_{i}$) about machine failure rates. The rest of the parameters ($E_{i},\;C_{E_{i}},\;P_{Fi},\;C_{F_{i}}$) are objective variables.

MES has no records of stage information, making it difficult to know stage-related parameters. In the case study, according to the experience of job shop operators, we made an assumption that the average duration of the preparing stage $t_{S_{i1}}$ is 1 h, after which raw materials are charged to the machine. The energy consumption profile of the investigated machine is estimated by machine power parameters and by consumed energy measured by a power meter. Each type of product has a specific power profile. Until now, all required parameters for Equations (6)–(9) to calculate energy cost $C_{E_{i}}$ are available.

**Figure 4:**
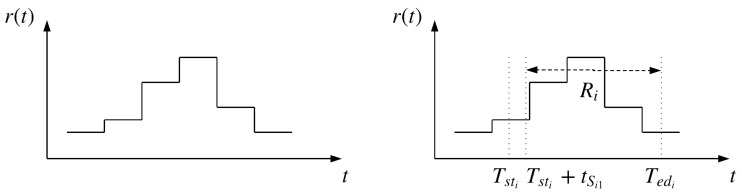
Retrieval of failure rate vector $R_{i}$ from the failure rate function.

The function of the failure rate between two adjacent maintenances r(t) is calculated using the average effect of maintenances, where the influences of maintenances f(t) are accumulated and normalized. *B* is a discretized representation of r(t). The procedure of retrieving $R_{i}$ is explained as follows:

In Figure 4, r(t) is the function of failure rate over time, $b_{k}=r\left(T_{pm}+k\right)$. Knowing $T_{st_{i}}$, $T_{ed_{i}}$, $t_{S_{i1}}$ (by our assumption $t_{S_{i1}}=1$) and the time of the previous maintenance $T_{pm}$, $R_{i}$ can be detected.

With all the required parameters known from the abovementioned procedure, Equations (12)–(14) are used for calculating failure cost. For the objective function in Equation (16), both $\omega_{1}$ and $\omega_{2}$ are set to 1.

### 5.2. Simulation Settings

Empirical MES records are also used as test cases of EFACPS. Before performing the proposed IGA, various settings of the experiment were made:

1. The concerned waiting job set in the experiment is a subset of all finished jobs in MES records. Derived from a fixed date range (from 2016-11-03 06:00:00 to 2016-11-07 17:00:00), 8 jobs were investigated in the case study. Details are provided in Table 3.
2. Corresponding Belpex RTP data have the same date range as the concerned waiting jobs.
3. The time step in the schedule is one second.
4. Maintenances are fixed on Saturdays.
5. Randomly generated data are used instead of real production data for some job characteristics (raw material cost and power profile) according to the confidentiality agreement.

In Table 3, job $j_{i}$ with product type $p_{i}$ has unit raw material cost $u_{p_{i}}$ following the distribution U(0.05,0.10)€/kg (from data source [35,36]) and power profile $g_{p_{i}}$ following the distribution U(0.08,0.15)MW (from data source [40]).

**Figure 5:**
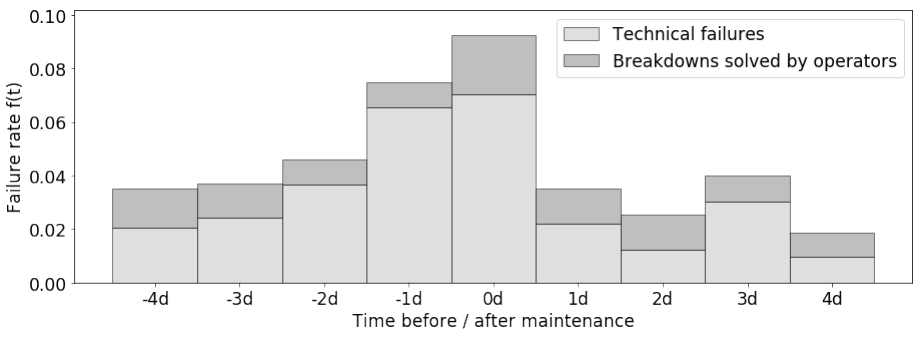
Failure rate as a function of time before and after maintenance (0d = maintenance day, −x d = x days before maintenance, +xd = x days after maintenance).

We used the procedures presented in Section 5.1 to calculate the average effect of maintenance f(t). The result in reality is shown in Figure 5, based on 90 maintenance records during 1 year.

In Figure 5, the failure rate f(t) consists of technical failures and small breakdowns solved by operators ($M_{Rr}$). Replacement maintenance ($M_{Re}$) is performed on day 0. As mentioned in Section 3.1.3, $M_{Rr}$ can not prevent the machine slowly falling into a worse health state; instead, $M_{Re}$ can restore the machine to a better health state. Therefore f(t) slowly increases during the days before maintenance (negative days) and sharply decreases on the day after maintenance (1 d), then keeps growing in the following days (other positive days).

With the average effect of maintenance f(t), we can calculate the failure rate of each day. For instance, 2016-11-04 is Thursday, which is 5 days after and 2 days before a maintenance day (Saturday); therefore, its failure rate $r=max[f_{1}\left(5\right),f_{2}\left(2\right)]$.

Parameters for IGA are tuned as follows: The population size α is set as 8; each generation has 8 candidate solutions. The maximal generation size $n_{2}$ is 200; therefore, the IGA will iterate the evolution for 200 generations. In each iteration, two chromosomes are selected and sorted by their fitness. The *winner* is retained to the next generation as the elitism, the *loser* is modified by genetic operations. The crossover rate ($p_{1}=60\%$) and mutation rate ($p_{2}=80\%$) are fixed. These high rates indicate that search points spread out among the solution space [41].

### 5.3. Results and Discussions

**Figure 6:**
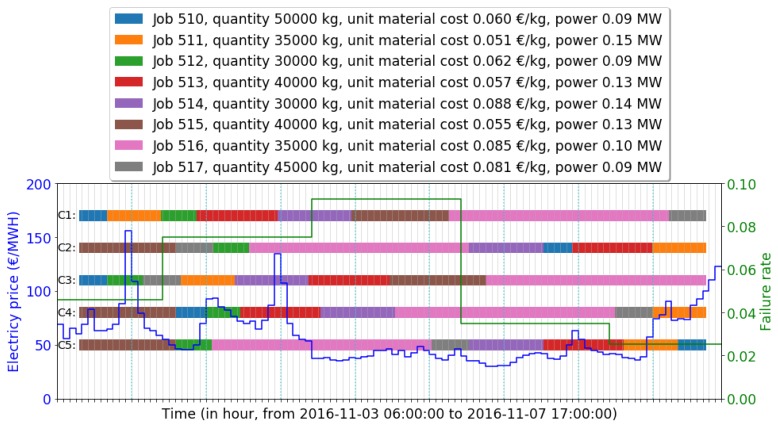
Visualization of the original schedule and candidate schedules with real-time (RTP) electricity price from Belpex and hourly dependent failure rate.

The near-optimal schedule found by IGA was compared with the original schedule and other candidate schedules: The single objective schedule and shortest job first (SJF) schedule. Figure 6 visualizes the original schedule and the candidate schedules with energy price and failure rate in the selected date range. Schedules are presented in continuous bar plots with different colors. Real-time electricity price changes hourly but remains the same within one hour, marked in blue. The failure rate is derived from previous sections, marked in green. The detailed comparison results of investigated schedules are provided in Table 4.

All candidate schedules start at 2016-11-03 09:30:51, which is the start time of job 510 in the original schedule C1. The near-optimal schedule C2 found by the IGA decreases both the energy cost and failure cost, saving 23.24% of total cost. The near-optimal schedule is further compared with other schedules using different policies. The candidate schedule C3 uses a classical policy, shortest job first (SJF), increasing 4.66% of the total cost than C1. Schedules considering a single objective are also inspected: The candidate schedule C4 minimizes the energy cost, saving 5.09% of energy cost and 5.77% of total cost. The candidate schedule C5 minimizes failure cost, saving 24.90% of failure cost and 23.21% of total cost. Among all candidate schedules, C2 has the lowest total cost.

A benefit of our proposed model is the flexibility to apply the schedules in actual production. Occurrences of short spare time (<1 h) on the machine do not conflict with our assumption that the machine is kept busy when there are remaining jobs. A slight left or right shift of a job will not affect the cost of a schedule. For instance, in C1, job 511 is scheduled to start at 2016-11-03 14:05:33 and to finish at 2016-11-03 22:47:09. The maximum left shift of job 511 is 2016-11-03 14:00:00 (start time). The maximum right shift is 2016-11-03 22:59:59 (end time). Candidate schedules also have such characteristics to accept shifts. For instance, the investigated schedules in this section have the maximum left shift to 2016-11-03 09:00:00 and the maximum right shift to 2016-11-07 14:59:59.

The scheduler can decide the weight of each objective in case there are special requirements or constraints. Huge consumers of electricity always sign contracts with providers, negotiating an agreement of low price for a certain amount of electricity [42]. Suppose the company has to pay extra fees for overuse of electricity: The scheduler adjusts $\omega_{1}$ to 15 without changing other parameters. The comparison results of candidate schedules after such a modification are provided in Table 5.

The provided IGA obtains the best result (saving 13.14% of total cost) among all candidate schedules. Experimental results also indicate that the studied EFACPS case of 8 jobs in this section has fewer opportunities for energy saving than for failure reduction. The failure model contributes more to the optimization than the energy model for this specific case. Before applying the IGA to actual EFACPS problems, the performance of the algorithm was investigated.

#### 5.3.1. Convergence Analysis

Since random sampling is used in the initialization step (see Section 4.1) of IGA, CGA, and RCA, a Monte Carlo simulation (MCS) is suitable to analyze the result and the performance of algorithms [43]. The studied case is an EFACPS of size 8 ($n_{1}$ = 8), having 8! = 40,320 possible candidate schedules. The MCS is set to run 50 times. Each simulation evaluates 207 candidate schedules, with 10,350 schedules in total. For a reasonable comparison, the search space of CGA and RCA should have the same size. Therefore, CGA has same paremeters ($n_{1},\;n_{2},\;\alpha ,\;p_{1},\;p_{2}$) as IGA. Each simulation of RCA runs 207 iterations for random choice.

**Figure 7:**
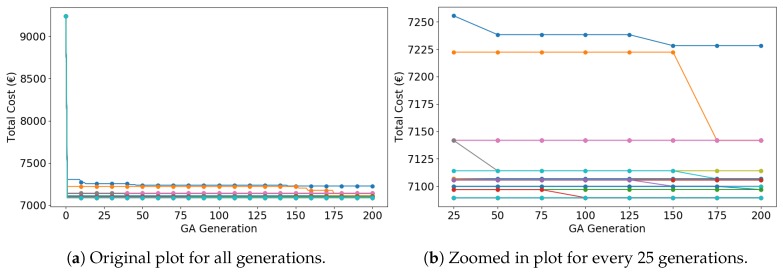
Total cost as a function of IGA generations (50 curves of 50 simulations).

Figure 7 depicts the IGA search trend of MCS. Figure 7a contains 50 curves; each curve corresponds to one simulation. Despite the consensus that GA produces a near-optimal solution [44], MCS with sufficient trails can provide an optimal solution. Shown in Figure 7b, the algorithm quickly converges to relatively good solutions in the early (<25) generations, with the cost decreasing from 9235.86 € to fewer than 7250 €. From 25 to 175 generations, the algorithm steadily converges to the optimal (or near-optimal) solutions. After 175 generations, the algorithm remains stable.

Due to the introduced features of memory, distance evaluation, and random technique, coupled with the conspicuously applied evolution strategy, IGA converges significantly faster than CGA, whose search trend is presented below.

**Figure 8:**
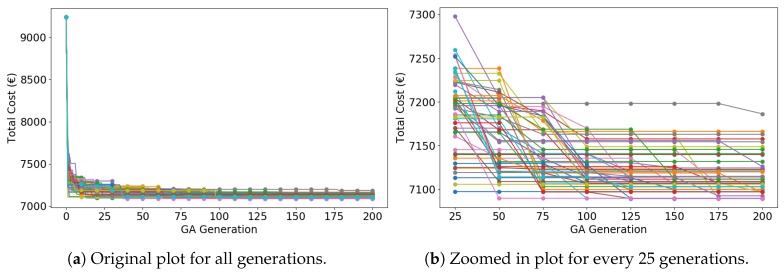
Total cost as a function of conventional genetic algorithm (CGA) generations (50 curves of 50 simulations).

In Figure 8, CGA needs at least 50 generations to obtain a relatively good solution (fewer than 7250 €), which is 25 generations slower than IGA. Throughout some simulations, CGA does not even converge in 200 generations. Duplicate search and trapping into local optima decline the convergence speed.

**Figure 9:**
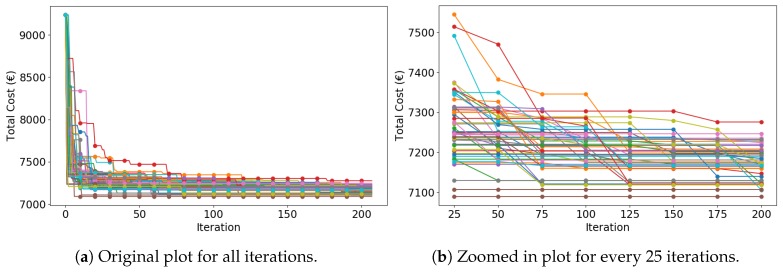
Total cost as a function of random choice algorithm (RCA) iterations (50 curves of 50 simulations).

The search trend of RCA is also provided in Figure 9. To get a relatively good solution (fewer than 7250 €), CGA needs at least 75 iterations. Therefore, it has the lowest convergence speed among the three algorithms. Nonconvergence exists in some simulations as well.

An important discussion point is the configuration of parameters when applying IGA. Different settings of parameters lead to distinct search trends, but a good parameter setting can make the algorithm converge faster. Because of random initialization, the same settings can also result in different search trends. In our experiment, this was avoided by giving a fixed random seed.

Regardless of other parameters, a larger generation (iteration) size always provides a better result. However, the scheduler is encouraged to view the search trend and apply other parameter tuning techniques. For instance, if the algorithm remains stable for more than 50 generations, it is considered already converged.

#### 5.3.2. Performance Evaluation

The provided algorithms (IGA, CGA, and RCA) are randomized optimization algorithms with arbitrary input, output, and performance. The graphical representations of 50 simulations in the previous section facilitate the understanding of how the algorithms behave when applied to the studied case of 8 jobs. The convergence speed of IGA (25 generations) is 3 times faster than that of RCA (75 generations) and 2 times faster than that of CGA (50 generations). In this section, statistical analysis was performed for further comparison.

**Figure 10:**
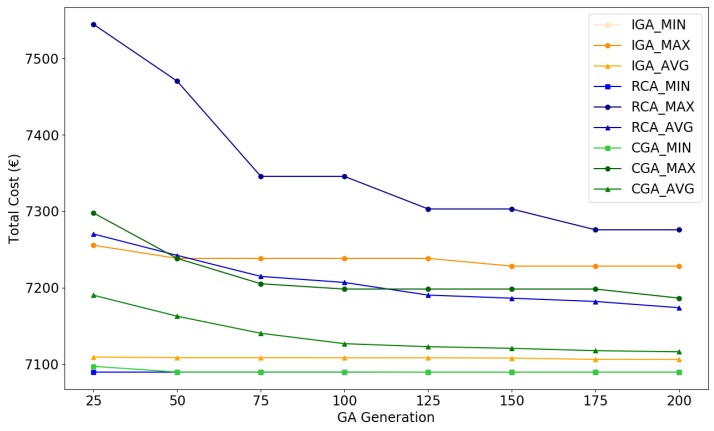
Minimum, maximum, and average of total cost as a function of generations of IGA, CGA, and RCA.

Figure 10 visualizes statistical indicators (minimum, maximum, average) of total cost during evolutions of IGA, CGA, and RCA.

In Figure 10, algorithms and statistical indicators are compared and distinguished using different colors and shapes. The averages are marked using triangles. In each iteration, RCA has the highest average cost, followed by CGA, while IGA has the lowest average cost. Therefore, IGA has the best average performance in the three algorithms. Both CGA and RCA have a decreasing average cost with the growth of iteration numbers, indicating the feasibility of these algorithms. The maxima are marked using circles, reflecting the worst performance of algorithms. All algorithms have a decreasing maximum cost when the iteration number increases. In each iteration, RCA has the highest maximum cost, whereas CGA and IGA have a relatively low maximum cost. The minima are marked using squares, representing the best performance of algorithms. All algorithms have the same minimum cost after 50 iterations. Numbers of simulations converged to the maximum cost, the minimum cost, and other cost values at iteration 200 are also provided in Table 6: For IGA, 17 in 50 simulations converge to the minimum. For CGA, this number reduces to 4. For RCA, only 1 simulation converges to the minimum.

**Figure 11:**
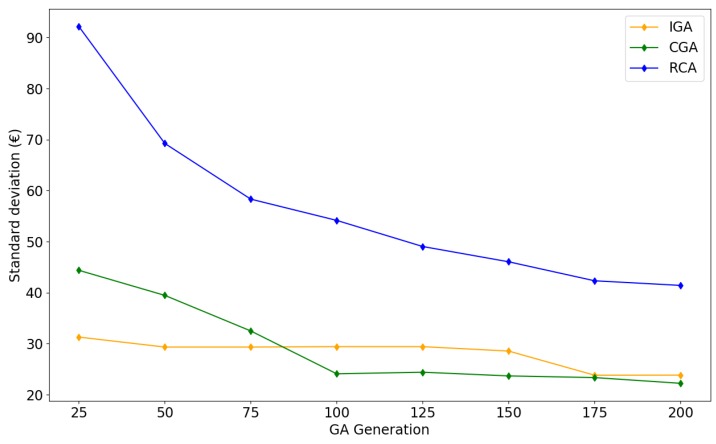
Standard deviation of total cost as a function of generations of IGA, CGA, and RCA.

The standard deviations (SD) of costs are calculated in Figure 11. All algorithms have decreasing SDs as the iteration number increases. In each iteration, RCA has the largest SD, followed by CGA. IGA has a relatively small SD, indicating that the simulation results of IGA do not have a large variation or dispersion. The SD of CGA decreases in early generations (<100) and remains steady afterwards.

The convergence summary and the statistical indicators from Table 6 support our inference that IGA has the best performance among the three provided algorithms: In 50 experimental results of MCS, IGA has the lowest average cost and a relatively small SD. Furthermore, IGA are more easily to converge to the minimum than the other two algorithms.

The coverage ratio [45] for the simulation results of RCA, CGA, and the IGA was also examined. Defined in Equation (17), the coverage ratio C(A,B) represents the number of points in set *B* dominated by set *A* over the total number of points.

(17) $$C\left(A,B\right)=\frac{\left|\{x\in B\;|\;\exists y\in A:y\;dominates\;x\}\right|}{\left|B\right|}$$

In our case, ydominatesx is defined if point *y* has lower or equal cost than point *x*. The summary of the coverage ratio between simulation results is presented in Table 7; a higher ratio of C(A,B) implies a better performance of *A* over *B* in the perspective of result coverage. C(IGA,CGA) and C(IGA,RCA) remains 1 in all iterations, indicating that IGA can always find an equal or better result than the other two algorithms. C(CGA,IGA) and C(RCA,IGA) vary in different generations. C(CGA,RCA) and C(RCA,CGA) change with the growth of iteration numbers, but in the end, C(CGA,RCA) converges to 1. Therefore, CGA can find an equal or better result than RCA when the iteration number increases.

#### 5.3.3. Complexity Analysis

The detailed procedure of the provided IGA is explained in Algorithm 3, where complexity analysis is conducted on each stage. We investigated the algorithm in the worst case, where each stage requires the longest time and largest space. Afterwards, the asymptotic upper bound [46] of time and space complexity of the algorithm was given.

The provided IGA was implemented in Python using specially designed data structures to improve the efficiency as possible. In the initialization stage, the time consumption for random generation is O(1), and the corresponding space consumption is $O(\alpha *n_{1})$. In the elitism selection stage, the time for creating sub_pop is O(1), for fitness value sorting is O(2), and the corresponding extra space requirement is $O(2*n_{1})$. In the crossover stage, the loser is replaced with DNAs from the original chromosome and the winner, where the time requirement is $O(n_{1})$ with no more space requirement. In the mutation stage, random choice and swap of positions demand O(1) time and no extra space. Distance evaluation has the time complexity of $O(n_{1})$ and space complexity of $O(2*n_{1})$. In the memory search stage, the time and space requirement is O(β∗α), where β is the size indicator of memory, a self-defined constant depending on the workstation running the algorithm. Finally, retrieving the best cost *P* and candidate schedule *C* requires $O(n_{1})$ time with no extra space.

To sum up, the time complexity of IGA is $O(n_{1}*n_{2})$ because of the outer loop of $n_{2}$ iterations. Therefore, the corresponding asymptotic upper bound of time complexity of IGA is $O(n^{2})$. The space complexity is O(n).

### 5.4. Stress Test

The efficiency of the provided IGA is further investigated in this section on a larger problem. On a normal PC (i7 CPU, 16G RAM) released in 2017, for $n_{1}=1122$ (number of jobs from 2016-01-19 14:00:00 to 2017-11-15 00:00:00, records of 2 years), $n_{2}=200$, and β=5, the algorithm requires 77.48 s to obtain a near-optimal schedule with the corresponding cost; each iteration takes 0.39s. The original schedule has the energy cost of 59,956.10 € and the failure cost of 1,228,990.41 €, in total 1,288,946.51 €.

**Figure 12:**
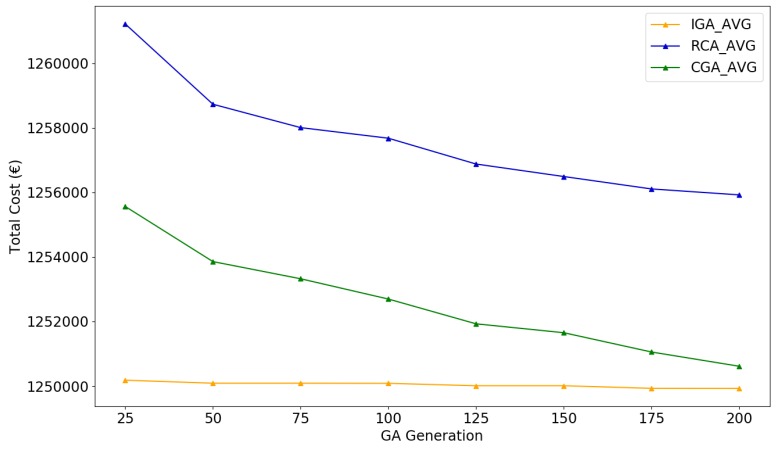
Average of total cost as a function of generations for IGA, CGA, and RCA on 1122 waiting jobs.

The performance of IGA, CGA, and RCA on such large problem scale was also analyzed using MCS. The number of simulations was set as 50; therefore, each algorithm was executed for about one hour to search for a near-optimal solution. Certainly for large-scale problems, the limited number of simulations cannot ensure that the IGA finds an optimal solution. Schedulers are encouraged to run more simulations for a potentially better result. Same as in Section 5.3.2, the convergence summary and the statistical indicators are presented in Figure 12 and Table 8.

Experimental results showed that after one hour’s execution, IGA can provide a near-optimal candidate schedule with 3.03% total cost saving in average compared to the original schedule, which is the lowest in the three provided algorithms (CGA 2.97%, RCA2.56%). Considering both Figure 12 and Table 8, IGA has a rapid convergence speed (fewer than 25 generations) to near-optimal schedules with the lowest average cost and is also more likely to converge to the schedule with the minimum cost (saving 3.17%).

## 6. Conclusions

In this paper, the energy- and failure-aware continuous production scheduling problem at the unit process level was investigated. The research put forward a coupled model of energy and failure cost and provides an improved genetic algorithm to solve it. The IGA was implemented in Python with the time complexity of $O(n^{2})$ and the space complexity of O(n). The algorithm was efficient to search for a near-optimal schedule with volatile energy prices and maintenance-dependent machine failure rates. Real industrial cases from a large pasta manufacturer were studied using the provided algorithms. Compared to an original schedule from empirical records (8 jobs in 5 days), the IGA provided a near-optimal schedule saving 23.24% of total cost. For another larger case (1122 jobs in 2 years), the IGA also found a near optimal solution, saving 3.17% of total cost. The results of Monto Carlo simulations indicate that IGA converges 2 times faster than CGA and 3 times faster than RCA and can always obtain better solutions within limited time and iterations.

Future research could further improve in several directions. Similar to other continuous production systems, like textile dyeing [8], steel-making [22], and construction [23], pasta manufacturing is both time-sensitive and quality-sensitive, with strict constraints of the food industry. The actual industrial environment is much more complicated than the background of the investigated EFACPS problem in this study, with special requirements of suppliers, customers, operators, machine configurations, and product status. The current study has limitations caused by the adopted assumptions in the modeling stage as a consequence of the multivariate production environment. For instance, the cost of maintenances is neglected in this study, but in reality, they are expensive. Additionally, the machine is not obliged to keep busy in continuous manufacturing, since idle periods are allowed between working periods in the face of small breaks, like the shortage of raw materials or the shift of operators. The proposed method should be further extended to multiobjective problems and more complicated machine environments. Modeling and algorithm design should also take into consideration the availability of data sources or actively make use of sensors and controllers.

In conclusion, this paper puts forward a new perspective of energy and failure management in continuous production scheduling and provides an improved genetic algorithm as an efficient solution with better performance than conventional genetic algorithms.

## Figures and Tables

**Table 1:** Parameters used for problem formulation.

Parameter	Description
*J*	set of waiting jobs with ID {1,2,…,N}
$j_{i}$	job with index *i*
*N*	number of waiting jobs
*P*	set of product types
*M*	number of possible product types
$p_{i}$	product type of $j_{i}$
$q_{i}$	objective quantity of $j_{i}$
$v_{p}$	unit production speed of product type *p*, where p∈P
$v_{p_{i}}$	unit production speed of $j_{i}$, whose product type is $p_{i}$
$t_{i}$	production duration of $j_{i}$
$T_{st_{i}}$	start time of $j_{i}$
$T_{ed_{i}}$	end time of $j_{i}$
$S_{i}$	set of processing stages of $j_{i}$
$T_{S_{i}}$	set of durations of stages of $j_{i}$
$t_{S_{ki}}$	duration of *k*th stage of $j_{i}$
$P_{s}$	power consumption of stage *s*
$E_{i}$	energy consumption of processing $j_{i}$
$C_{E_{i}}$	energy cost of processing $j_{i}$
$D_{i}$	vector of energy price
$N_{D_{i}}$	size of $D_{i}$
*H*	set of machine health states
$h_{k}$	*k*th state ranked with failure rate
$M_{Rr}$	real-time response maintenance (maintenance type)
$M_{Re}$	replacement maintenance (maintenance type)
π	a schedule
r(t)	machine failure rate changing over time *t*
$T_{pm}$	time stamp of a particular maintenance
*B*	vector of failure rate after maintenance
$b_{k}$	failure rate of *k*th hour after maintenance
$R_{i}$	vector of failure rate after raw material charged on the machine
$P_{Fi}$	probability of failure occurrence during production of $j_{i}$
$u_{p_{i}}$	unit raw material cost of product type $p_{i}$
$C_{F_{i}}$	failure cost of $j_{i}$

**Table 2:** Parameters in data records.

Record	Parameters
OIR	$J,\;j_{i},\;N,\;P,\;M,\;p_{i},\;q_{i},\;u_{p_{i}}$
OPR	$v_{p},\;t_{i},\;T_{st_{i}},\;T_{ed_{i}}$
MSR	$H,\;h_{k}$
MER	$T_{pm}$
BELPEX	$D_{i},\;N_{D_{i}}$

**Table 3:** Waiting jobs for scheduling.

ID	Product Type	Objectif Quantity	Start Time	End Time	Unit Material Cost	Power
510	FF011501	40,000	2016-11-03 09:30:51	2016-11-03 14:05:29	0.055	0.13
511	FF082005	45,000	2016-11-03 14:05:33	2016-11-03 22:47:09	0.081	0.09
512	FF025201	30,000	2016-11-03 22:47:15	2016-11-04 04:32:20	0.062	0.09
513	FF027216	35,000	2016-11-04 04:32:23	2016-11-04 17:29:46	0.085	0.10
514	FF049361	30,000	2016-11-04 17:29:50	2016-11-05 05:27:33	0.088	0.14
515	FF049390	50,000	2016-11-05 05:27:41	2016-11-05 21:23:04	0.060	0.09
516	FF174906	40,000	2016-11-05 21:23:07	2016-11-07 08:46:27	0.057	0.13
517	FF044101	35,000	2016-11-07 08:46:35	2016-11-07 14:49:42	0.051	0.15

**Table 4:** Comparison between the original schedule and the candidate schedules.

**ID**	**Case**			**Sequence**		
C1	Original schedule from historical record			[510, 511, 512, 513, 514, 515, 516, 517]		
C2	Near-optimal schedule using IGA ($\omega_{1}=1,\omega_{2}=1$)			[515, 517, 512, 516, 514, 510, 513, 511]		
C3	Schedule using shortest job first policy			[510, 512, 517, 511, 514, 513, 515, 516]		
C4	Schedule minimizing energy cost			[515, 510, 512, 513, 514, 516, 517, 511]		
C5	Schedule minimizing failure cost			[515, 512, 516, 517, 514, 513, 511, 510]		
**ID**	**Energy Cost**	**Failure Cost**	**Overall Cost**	**Energy Saving**	**Material Saving**	**Total Saving**
C1	655.69	8580.17	9235.86	0%	0%	0%
C2	641.17	6448.29	7089.46	2.21%	24.85%	23.24%
C3	666.53	8999.98	9666.51	−1.65%	−4.89%	−4.66%
C4	622.30	8080.37	8702.67	5.09%	5.83%	5.77%
C5	648.42	6444.06	7092.48	1.11%	24.90%	23.21%

**Table 5:** Comparison between the original schedule and the candidate schedules.

**ID**	**Case**			**Sequence**		
C6	Original schedule from historical record			[510, 511, 512, 513, 514, 515, 516, 517]		
C7	Near-optimal schedule using IGA ($\omega_{1}=15,\omega_{2}=1$)			[515, 517, 512, 516, 514, 510, 513, 511]		
C8	Schedule using shortest job first policy			[510, 512, 517, 511, 514, 513, 515, 516]		
C9	Schedule minimizing energy cost			[515, 510, 512, 513, 514, 516, 517, 511]		
C10	Schedule minimizing failure cost			[515, 512, 516, 517, 514, 513, 511, 510]		
**ID**	**Energy Cost**	**Failure Cost**	**Overall Cost**	**Energy Saving**	**Material Saving**	**Total Saving**
C6	9835.40	8580.17	18,415.57	0%	0%	0%
C7	9527.27	6467.66	15,994.93	3.13%	24.62%	13.14%
C8	9997.90	8999.99	18,997.89	−1.65%	−4.89%	−3.16%
C9	9334.48	8080.37	17,414.86	5.09%	5.83%	5.43%
C10	9726.34	6444.06	16,170.40	1.11%	24.90%	12.19%

**Table 6:** Convergence summary and statistical indicators for 50 Monte Carlo simulations (MCS) of IGA, CGA, and RCA at iteration 200.

**Algorithm**	**Converge to Maximum**	**Converge to Minimum**	**Converge to Others**	
IGA	1	17	32	
CGA	1	4	45	
RCA	1	1	48	
**Algorithm**	**Maximum Cost**	**Minimum Cost**	**Average Cost**	**Standard Deviation**
IGA	7228.25	7089.46	7106.10	23.84
CGA	7186.35	7089.46	7116.11	22.23
RCA	7275.73	7089.69	7173.80	41.42

**Table 7:** Coverage ratio analysis for IGA, CGA, and RCA.

Generation	C(IGA, CGA)	C(CGA, IGA)	C(IGA, RCA)	C(RCA, IGA)	C(CGA, RCA)	C(RCA, CGA)
25	1	0.62	1	0.68	0.98	1
50	1	0.68	1	0.68	1	1
75	1	0.68	1	0.68	1	1
100	1	0.66	1	0.66	1	1
125	1	1	1	0.66	1	0.96
150	1	1	1	0.66	1	0.96
175	1	1	1	0.66	1	0.92
200	1	1	1	0.66	1	0.92

**Table 8:** Convergence summary and statistical indicators for 50 MCS of IGA, CGA, and RCA on 1122 waiting jobs at iteration 200.

**Algorithm**	**Converge to Maximum**	**Converge to Minimum**	**Converge to Others**	
IGA	1	6	43	
CGA	1	1	48	
RCA	1	1	48	
**Algorithm**	**Maximum Cost**	**Minimum Cost**	**Average Cost**	**Standard Deviation**
IGA	1,258,670.98	1,248,039.40	1,249,926.71	2419.96
CGA	1,255,047.27	1,241,847.12	1,250,612.83	2698.97
RCA	1,261,961.80	1,251,042.40	1,255,922.92	2692.98
